# Rapamycin Ameliorates Radiation-Induced Testis Damage in Mice

**DOI:** 10.3389/fcell.2022.783884

**Published:** 2022-04-25

**Authors:** Juan Yang, Rui Xu, Yingying Luan, Hancheng Fan, Shuo Yang, Jun Liu, Huihong Zeng, Lijian Shao

**Affiliations:** ^1^ School of Public Health, Jiangxi Provincial Key Laboratory of Preventive Medicine, Nanchang University, Nanchang, China; ^2^ Department of Blood Transfusion, Affiliated Hospital of Chengde Medical College, Chengde, China; ^3^ School of Basic Medicine, Nanchang University, Nanchang, China; ^4^ The Department of Urology, Huashan Hospital, Fudan University, Shanghai, China

**Keywords:** radiation, testis, mTORC1, cell proliferation, apoptosis

## Abstract

Male infertility is an important problem in human and animal reproduction. The testis is the core of male reproduction, which is very sensitive to radiation. The decline of male reproductive ability is a common trend in the world. Radiation is a physical factor leading to abnormal male reproductive function. To investigate the potential mechanisms of testicular damage induced by radiation and explore effective strategies to alleviate radiation-induced testis injury, C57BL/6 mice were irradiated with 8.0 Gy of X-ray irradiation. Testis and epididymis were collected at days 1, 3, and 7 after radiation exposure to analyze spermatogonia and sperm function. The results showed that radiation significantly destroyed testicular structure and reduced the numbers of spermatogonia. These were associated with mTORC1 signaling activation, decreased cellular proliferation and increased apoptotic cells in the irradiated testis. Rapamycin significantly blocked mTORC1 signaling pathway in the irradiated testis. Inhibition of mTORC1 signaling pathway by rapamycin treatment after radiation could significantly improve cell proliferation in testis and alleviate radiation-induced testicular injury after radiation exposure. Rapamycin treatment benefited cell survival in testis to maintain spermatogenesis cycle at 35 days after irradiation. These findings imply that rapamycin treatment can accelerate testis recovery under radiation condition through inhibiting mTORC1 signaling pathway.

## Introduction

Radiation, especially ionizing radiation, exists in the contaminant environment and injures cells, tissues, and organs. Meanwhile, radiation is also widely used in clinic as medical technical means. Radiotherapy is an effective treatment for malignant tumors. More than 70% of tumor patients need to receive different doses of radiotherapy. For example, radiotherapy is applied to treat various kinds of cancers including lung, cervical, rectal, prostate, and breast cancers (Smit et al., 2011; Valicenti et al., 2013; Klopp et al., 2014; Minicozzir et al., 2014; Rodrigues et al., 2015). It is well known that intestinal and hematopoietic cells are sensitive to radiation. Ionizing radiation leads to hematopoietic system damage through Puma-mediated apoptosis imbalance (Shao et al., 2014). Meanwhile, ionizing radiation can cause massive loss of intestinal crypt stem cells and cause intestinal damage (Bhanja et al., 2018). Many approaches are under exploring to mitigate radiation-induced intestinal and hematopoietic damage. However, reproductive system, particularly male reproduction, is easily injured by radiation exposure.

Spermatogenesis of testis is a strict and orderly process of spermatogenic cells. The proliferation, renewal and differentiation of spermatogonia are important steps of spermatogenesis. Undifferentiated spermatogonia are usually found in the area of the basement membrane adjacent to the interstitial tissue and the vascular system. Spermatogonia undergo cell division and differentiation to form sperms, which are stored in the epididymis. Undifferentiated, dividing and immature cells, such as intestinal epithelial cells and hematopoietic cells, are more radiosensitive than fully differentiated, nondividing and mature cells. Radiosensitive cells easily die, leading to various tissue dysfunction and pathological changes. Radiation-induced tissue damage is mainly through the immediate formation of free radical/reactive oxygen species (ROS). The antioxidant system can’t be fully loaded, leading to adverse reactions of male reproductive infertility. Oxidative stress (OS) has been identified as an important cause and common mechanism of many known and unknown causes of male infertility (Agarwal et al., 2021). At present, anti-radiation drugs approved by FDA, such as Amifostine and Filgrastim, have been used in clinical practice to improve the survival rate of patients exposed to lethal dose radiation (Citrin et al., 2010; Dumont et al., 2010; Koukourakis 2012). However, no effective countermeasure is available to ameliorate male infertility and gonadal dysfunction, which occur as side effects during radiotherapy. Therefore, radiation-induced reproductive toxicity severely affects the quality of life of cancer patients (Oermann et al., 2011; Thompson et al., 2018).

Reproductive system, especially in male, is one of the most sensitive organs to radiation exposure (Bonde 2010; Liu et al., 2015). Testis is mainly composed of germ cells, sertoli cells, and Leydig cells. The coordination and cooperation of various types of cells in the testis are the basis to ensure normal spermatogenesis and maintain spermatozoa count. Testis contains large numbers of seminiferous tubules, including all levels of spermatogonia, which is an important environment for spermatogenesis. Spermatogenesis occurs in seminiferous tubules of the testis in a highly orchestrated process, which eventually forms sperm in the testis (Singh and Jaiswal 2011). Proliferation of spermatogonia stem cells (SSCs) expands spermatogonia, which processes to meiosis and generates spermatozoa. Spermatogonia are the most sensitive to radiation in testis. Radiation on the testis may lead to a decrease in spermatogenic cells, a serious reduction in numbers of spermatozoa or even no sperm, leading to the emergence of infertility (Trost and Brannigan 2012; Fukunaga et al., 2018). Irradiation-induced SSC injures cause long-term dysfunctional male gonads. To protect damaged male reproductive system under irradiation conditions, it is immediately needed to develop novel mitigators, which will benefit for those populations exposed to unintended radiation and unclear accident (Hafer et al., 2010). Importantly, well-developed mitigators will benefit injured normal tissues and cells during radiotherapy, which will improve cancer treatment effects.

Rapamycin, also known as sirolimus, is a macrolide isolated from *Streptomyces hygroscopicus*. Rapamycin is a specific inhibitor of mTOR, through specifically binding to mTOR. By integrating extracellular signal inputs, amino acids and nutrients, mTOR complex plays crucial roles in regulating cell growth and metabolism. Dysregulated metabolic homeostasis and growth control often appear in diabetes, cancers and other diseases, respectively (Lu et al., 2021). Rapamycin is a potential cancer treatment drug and plays important roles in inhibiting the development of liver cancer to further understand targets of prevention and treatment of liver cancer (Lu et al., 2021). Additionally, few studies have shown that rapamycin is of great benefit in prolonging the life span of organisms (Dumas and Lamming 2020; Liu and Sabatini 2020). The beneficial effects of inhibiting mTOR signaling have been demonstrated through rapamycin administration initiating at mid- to late-life. Experimental data showed that rapamycin could improve both health-span and lifespan in mice even thought it was delivered late in life (Zhang et al., 2014). Amber E Kofman found that rapamycin can restore the normal expression of oxidative stress response genes in aging SSCs and improve the self-renewal ability of SSCs (Kofman et al., 2012). Long-term exposure to rapamycin can enlarge the SSC pool and increase the expression of Gfra1 and Ret in mouse testis (Hobbs et al., 2010). Thus far, no data is shown whether rapamycin could mitigate radiation-induced reproductive toxicity.

There are two different types of mTOR complexes, mammalian rapamycin target complex 1 (mTORC1) and mTORC2. mTORC1 is composed of mTOR, Raptor, mLST8 and two different inhibitory chaperones, PRAS40 and DEPTOR. The components of mTORC2 complex include mTOR, mLST8, Rictor, DEPTOR, mSIN1, and Protor1/2 protein (Yang et al., 2013). Rapamycin efficiently inhibits mTORC1 signaling but not mTORC2 signaling. Activation of mTORC1 phosphorylates downstream substrates: S6K kinase, 4EBP1 and transcription factor TFEB, to promote cell metabolism. The earliest and most characteristic substrates of mTORC1 are S6 kinase (S6K) and 4EBP1. 4EBP1 is eukaryotic translation initiation factor 4e (EIF4E) binding protein 1. mTOR activates S6K and inhibits 4EBP1 resulting in promoting translation and cell proliferation. Rapamycin interacts with FKBP12 in mammalian cells to form the FKBP12-rapamycin complex. The FKBP12-rapamycin complex docks with FRB domain to inhibit the activity of mTORC1 kinase. In *in vivo* study of SSC maintenance and downstream gene regulation of mTORC1, it was found that inhibition of mTORC1 not only upregulated the key genes essential for SSC self-renewal, but also increased the transcriptional levels of oxidative stress genes and down-regulated the genes related to growth and metabolism (Kofman et al., 2012). GDNF signal transduction and PLZF-mediated Redd1 activation antagonize mTORC1 promoting SSC self-renewal in mice. mTORC1 is a downstream factor of AKT/MAPK. Early resting SSCs are activated under the stimulation of AKT/MAPK, which then forms early and late progenitor cells under the action of mTORC1, generating differentiated spermatogonia to maintain normal spermatogenesis (Hobbs et al., 2010).

Radiation exposure-induced risks are inevitable among radiation-related workers and cancer patients. Efficient countermeasures need to be developed to protect individuals who may be exposed under radiation settings. Rapamycin has positive therapeutic effects on diabetes and cancers. An appropriate amount of rapamycin can restore the self-renewal ability of germ cells, but its role in radiation-induced testicular injury is not clear. In this regard, we hypothesized that rapamycin plays a protective role in SSCs injury induced by radiation. Therefore, the mouse testicular injury model was presently established by whole body X-ray irradiation. The morphological and functional damage of testis and the status of mRORC1 signal pathway were assessed. Rapamycin was used to inhibit mTORC1 signal pathway, so as to explore the protective effect and possible mechanism of rapamycin on testicular injury induced by X-ray. The present study will provide new ideas and strategies for the prevention and treatment of testicular injury induced by radiation.

## Materials and Methods

### Mice

All animals used in the experiment were 8-week-old C57BL/6J male mice purchased from Hunan SLAC Experimental Animal Center. The feeding environment of mice in 12 h each in dark and light. Food and water can be ingested freely. After a week of feeding in an adaptive environment, 84 mice (22 ± 2 g) were used in the experiment. All operations of animal handling are in accordance with the regulations of Nanchang University on Institutional Animal Care and Use Committee.

### Chemicals

Rapamycin (Rap) obtained from Meilun Biotechnology Co., Ltd., China was dissolved in 100% ethanol and prepared into 10 mg/ml stock solution, which was kept at −20°C. The stock solution is dissolved with 5% tween-80 and 5% PEG-400 to desired concentration and then used for injection. Alcohol and tween-80 are used to promote drug dissolution. PEG-400 is used to promote drug dissolution and maintain drug stability. The desired concentration of rapamycin is 4 mg/kg. The 5-bromo-2′-deoxyuridine (BrdU, Beijing Solarbio technology Co.,Ltd., China) of 100 mg/kg was injected into the animal 2 h before the sample was collected. The stock concentration of BrdU was 10 mg/ml and kept away from light.

### Irradiation

Four groups: non-irradiated group (CTL), irradiated group (X), rapamycin group (Rap), irradiation + rapamycin group (X + Rap), were randomly assigned to C57BL/6J mice. C57BL/6 mice were analyzed at 1, 3, 7, and 35 days after irradiation. There were six mice at each time point with a total of 72 mice. The mice were irradiated with 8 Gy X-rays by ElektaPrecise linear accelerator (2.28 Gy/min). Mice in the CTL group were not irradiated and administrated with vehicle. Mice in X group were irradiated and subcutaneously injected with vehicle after whole body irradiation. Mice in Rap group were not irradiated and treated with rapamycin. Mice in X + Rap group were irradiated and treated with rapamycin (4 mg/kg) subcutaneously at 6 h, 2, 4, and 6 days after irradiation.

### Sperm Count and Functional Test

#### Sperm Suspension

Bilateral epididymis was washed with saline, placed in an Eppendorf tube preheated with saline (1 ml) at 37°C, cut into pieces, and incubated in a constant temperature water bath at 37°C for 20 min. The sperm suspension was filtered with 70 µm cellular strainer, and the sperm suspension was ready for use.

#### Sperm Density

The sperm suspension was filled into a clean and improved abalone counting plate. All sperms (upper, lower, left, right, middle) were counted based on the sperm head. The spermatozoa were counted on the grid line according to the principle of counting up and down and left and right. Sperm density (10^6^/ml) = n × 50000 × dilution factors.

#### Sperm Motility

Sperm suspension was slowly filled into the improved abalone counting board. 5–10 visual fields were observed under high power microscope. 200 sperms were counted, and motility of sperms was graded (counted three times). According to the grading method recommended by WHO, sperm motility was divided into four grades: a, fast forward movement; b, slow or sluggish forward movement; c, non-forward movement, *in situ* movement; d, sperm immobility. Sperm motility (%) = (a+ b + c)/(a+ b + c + d) × 100%.

#### Sperm Survival Rate

The sperms were stained with eosin in five visual fields. Live sperms were non-stained and dead sperm stained with red. The numbers of live and dead sperm were counted under a microscope. Sperm survival rate (%) = numbers of live sperm/(numbers of live sperm + numbers of dead sperm) × 100%.

#### Sperm Morphology

The morphology of sperm was observed under a microscope after eosin staining for 1 h. 1,000 spermatozoa with intact structure were observed. The numbers of abnormal spermatozoa and normal spermatozoa were counted. Sperm morphology (%) = the number of normal spermatozoa/total number of spermatozoa × 100%.

### General Condition and Histopathological Examination of Testis

Testicular volume was measured, testes were weighed in order to obtain index of testis, and photos were taken before testicular fixation. The left testis was fixed with modified Davidson’s fixation solution and embedded in paraffin. 4.0 μm testicular sections were used for histological examination.

Immunohistochemical staining of antigen-antibody binding principle was used to detect the expression of PLZF (1:50, Santa Cruz Biotechnology, United States), PCNA (1:100, Boster, China), and pS6 (1:300, Cell Signaling Technology, United States). After the second antibody was incubated with the first antibody, the positive staining was colored with DAB staining, and the hematoxylin counterstaining was used to show the nucleus. PCNA was used to detect the proliferation of spermatogonia, PLZF marked spermatogonia stem cells, and pS6 reflected the effects of radiation on mTORC1 signaling in the testicular tissue.

PCNA, PLZF, and TUNEL apoptosis immunohistochemical staining positive cells were counted: 10 fields were randomly selected by high power of microscope (10 × 40). The number of positive cells in each seminiferous tubule were counted. The average numbers of positive cells per seminiferous tubule were counted. Integral optical density (IOD) calculation of pS6 positive protein: the software pro plus measures the IOD of 10 randomly selected visual fields under high power of microscope (10 × 40) to evaluate the expression of pS6.

### Detection of Activation of Mammalian Rapamycin Target Complex 1 Signal Pathway by Western Blotting

Testicular tissues of mice were frozen in liquid nitrogen until further use. The testicular tissue was cracked with RIPA buffer containing protease inhibitor (Applygen, Beijing, China). The supernatant of testicular tissue protein was obtained after centrifugation. The objective proteins and internal housekeeping gene protein were separated in SDS-PAGE. According to the molecular weight of the target proteins and housekeeping gene, the protein bands of pS6, S6, p-mTOR, mTOR, and β-actin were observed. The protein was transferred to the PVDF membrane in standard Tris-glycine transfer buffer, pH 8.3, containing 0.1% SDS. After transferring, the PVDF membranes were blocked for 2 h with 5% non-fat milk powder at room temperature. They were then incubated with anti-S6 (1:1,000, Cell signaling technology, United States), anti-phospho-S6 (1:2,000, Cell signaling technology, United States), anti-mTOR (1:2,000, Abcam, United Kingdom), anti-phospho-mTOR (1:2,000, Abcam, United Kingdom), anti-β-actin (1:2000, Cell signaling technology, United States) diluted in Western special diluent for primary antibodies (Wuhan Boster, China) overnight at 4°C. Membranes were then washed in TBST for 30 min, incubated with HRP conjugated secondary antibodies, diluted 1:10,000 (Beijing Trans, China) in TBST containing 5% non-fat milk powder. Membranes were resolved by chemiluminescence (Beijing TIANDZ, China). The results from western blot were biologically repeated three times. The band densities of pS6 and p-mTOR from western blot were quantitated using ImageJ software (http://rsbweb.nih.gov/ij) and calculated according to the S6 and mTOR band density, respectively.

### Detection of Apoptosis by Terminal-Deoxynucleotidyl Transferase Mediated Nick End Labeling Staining

Terminal-deoxynucleotidyl transferase mediated nick end labeling (TUNEL) apoptosis detection kit is used to detect the nuclear DNA breakage of tissue cells in the early stage of apoptosis and can accurately reflect the typical biochemical and morphological characteristics of apoptosis. Fluorescein-labeled dUTP connects to the 3′-OH end of DNA cleavage in apoptotic cells and specifically binds to luciferin antibody linked to horseradish peroxidase (HRP, horse-radish peroxidase), which reacts with HRP substrate diaminobenzidine (DAB) to produce a strong color reaction (dark brown). Finally, apoptosis in testis is observed and counted under microscope. Testicular sections were stained according to the methods and steps provided by TUNEL apoptosis detection kit.

### Detection of Genes Related to Proliferation and Apoptosis

RNA was extracted from testicular tissue cryopreserved in liquid nitrogen according to the steps of RNA extraction, RNA quality was measured and cDNA was synthesized by Thermo Scientific RevertAid First Strand cDNA Synthesis kit (Thermo, United States). Finally, the expression of Puma, Bak, Bax,Bcl-2, Bcl-xl, P57, P27, and Cyclin D2 was quantitatively detected by QIAGEN QuantiNova™ SYBR GREEN PCR Kit (QIAGEN, German). HPRT was used as a housekeeping gene. Primer sequences are available upon request.

### Statistical Analysis

The data were analyzed by GraghPadPrism6, and the measurement data were expressed by mean ± standard deviation. The data were analyzed by analysis of variance (ANOVA). Dunnett’s multiple comparisons test after one-way ANOVA and Sidak’s multiple comparisons test after two-way ANOVA methods were used for statistics. *p* < 0.05 indicates that the difference is statistically significant.

## Results

### Total Body Irradiation Induces Descending of Spermatogonia Cells, Sperm Quality, and Testis Coefficient

**FIGURE 1:**
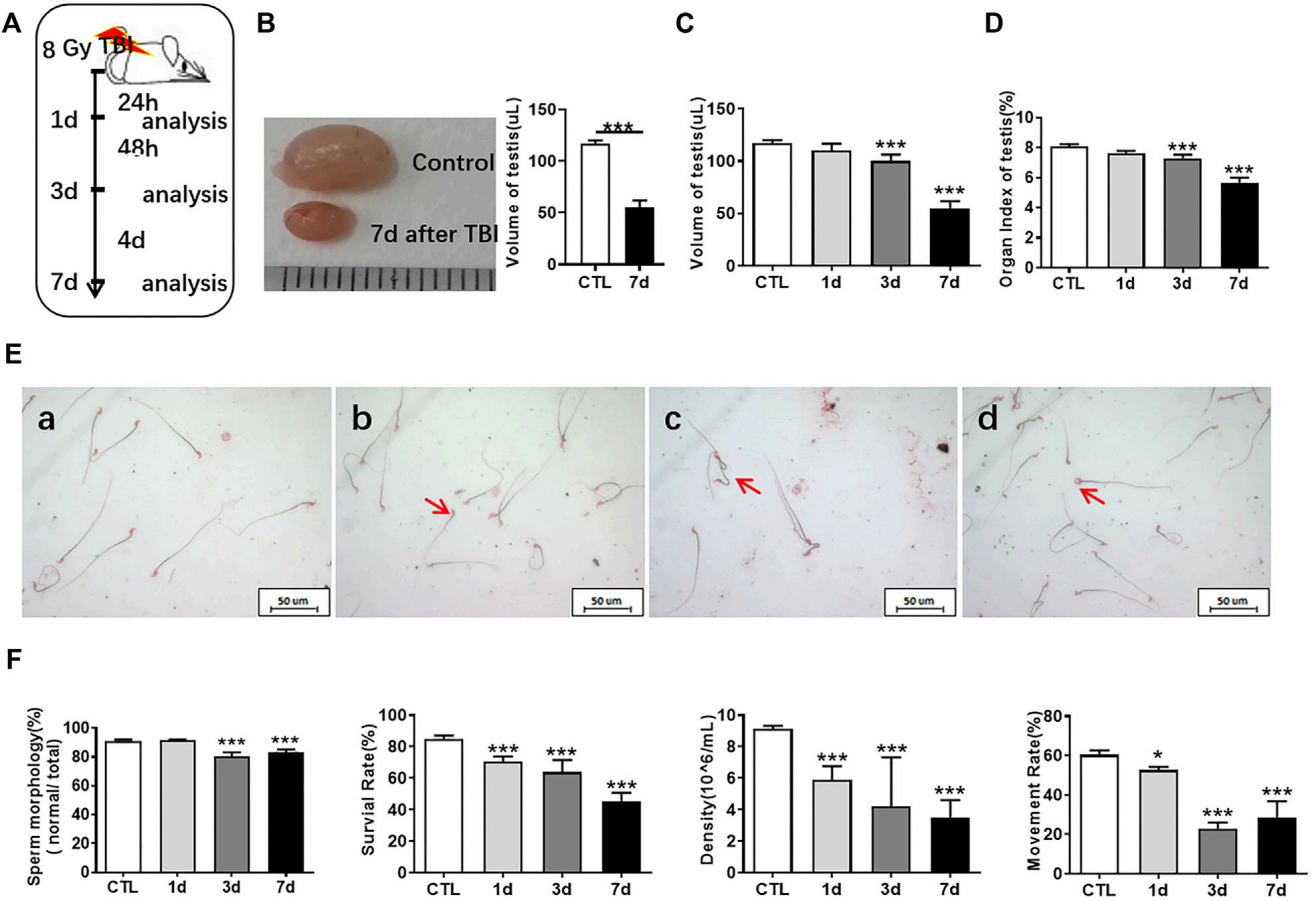
Radiation reduces testicular volume and sperm quality. **(A)** Experimental design. Testis and epididymis of 8-week-old C57BL mice were collected at 1, 3, and 7 days after whole body radiation exposure. **(B)** The testis of unirradiated mice and mice exposed to radiation for 7 days. **(C)** Testicular volume of mice at different time points after radiation exposure. **(D)** Organ index of testis at different time points after radiation exposure. **(E)** Sperm morphology. a, normal sperm; b, head unstereotyped sperm; c, tail folding sperm; d, double head deformed sperm. Bar = 50 μm. **(F)** Sperm quality parameters. From left to right, the sperm morphology, survival rate, concentration, and motility rate were shown. *p < 0.05 vs. CTL. **p < 0.01 vs. CTL. ***p < 0.001 vs. CTL. Dunnett’s multiple comparisons test was used after one-way ANOVA.

To explore the changes of mouse testis at different time points after whole body exposure to radiation, we measured the numbers of spermatogonia, sperm quality parameters and testis coefficient at 1, 3, and 7 days after whole body exposure (Figure 1A). As shown in Figure 1B, the size of testis at 7 days after irradiation was obviously smaller than that in the non-irradiated controls. In the overall appearance index of testis, the testicular volume did not change significantly at 1 day after radiation (Figure 1C), while the testicular coefficient significantly decreased at 3 and 7 days after radiation exposure (Figure 1D). Organ index of testis is the ratio of bilateral testicular weight to mouse body weight, which indirectly indicates the histopathological changes of testis.

Sperms are derived from the seminiferous epithelium in the testis. Abnormal sperm function may be caused by testicular damage induced by radiation. Sperm quality and testis coefficient can reflect the effects of radiation on testis. Sperm concentration indicates the amount of sperm formed by spermatogonia in the testis and eventually stored in the epididymis. The trend of spermatogonia reduction seems to be consistent with that of spermatozoa. The main manifestations of sperm deformities after radiation exposure were head and tail deformities, especially double heads, un-stereotyped and unhooked. The tail deformities were mainly double tails and tail folds (Figure 1E). The formation of sperm malformation may eventually be attributed to the whole process of spermatogenesis. The parameters of sperm quality include sperm density, sperm motility, sperm survival rate, and sperm morphology. The sperm quality decreased at 1, 3, and 7 days after irradiation, which showed that the sperm concentration significantly decreased, the sperm survival rate and motility rate significantly reduced, and the number of normal sperm remarkably decreased with the extension of time after radiation (Figure 1F).

**FIGURE 2:**
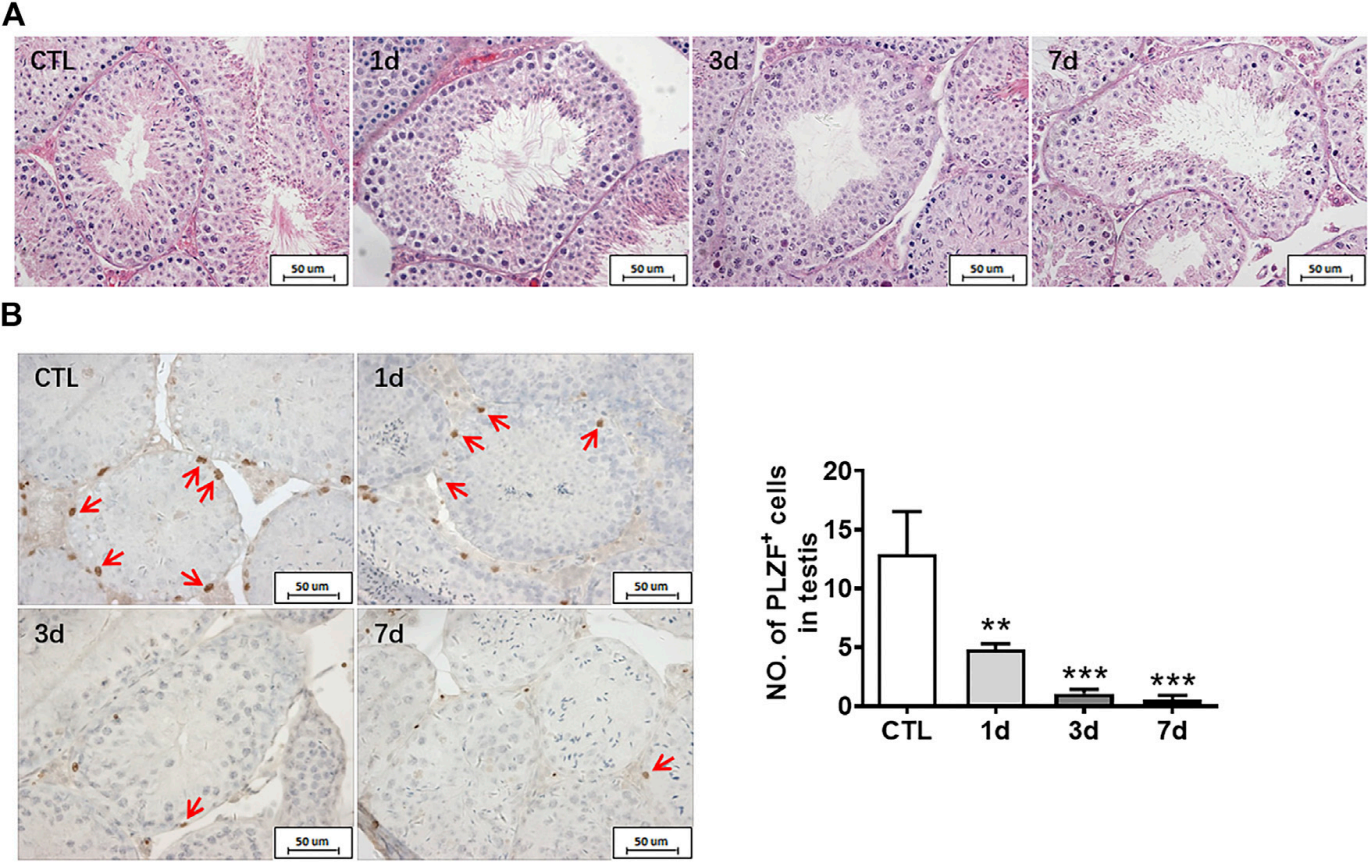
Radiation causes testicular injury and reduction of spermatogonial stem cells. **(A)** HE staining of testis. Bar = 50 μm. **(B)** PLZF immunohistochemical staining. PLZF immunohistochemical staining showed changes in the numbers of spermatogonia stem cells at different time points after whole body radiation exposure in mice (positive staining indicated with arrow). Numbers of PLZF^+^ cells were counted under light microscope and expressed as mean ± SD (right panel). Bar = 50 μm *p < 0.05 vs. CTL. **p < 0.01 vs. CTL. ***p < 0.001 vs. CTL. Dunnett’s multiple comparisons test was used after one-way ANOVA.

HE staining was used to observe the structure of seminiferous tubules in mouse testes, showing that the cells of seminiferous tubules in the unirradiated group are distinct and compact. In the radiation group, the arrangement of testicular seminiferous tubules was loose. The lumen became larger and more irregular in irradiated mice than that in normal controls. The spermatogenic cells decreased and arranged in disorder after radiation exposure. There were fewer intact spermatozoa in the lumen in the radiation group. The lumen became larger and more irregular along with fewer intact spermatozoa in the irradiated lumen when compared to the non-irradiated controls (Figure 2A).

To further measure the spermatogenic function of testicular tissue, the numbers of spermatogonia were assessed. Undifferentiated spermatogonia was labelled with PLZF antibody. The results showed that the numbers of PLZF positive cells significantly decreased at day 1, day 3, and day 7 after irradiation when compared to non-irradiated group (Figure 2B). The numbers of spermatogonia in the testis were decreased in a time dependent manner after radiation (Figure 2B). Taken together, radiation not only negatively affects the morphology of seminiferous tubules but also damages undifferentiated spermatogonia.

### Rapamycin Ameliorates the Reduction of Spermatogonia Cells and Sperm Quality Under Whole Body Irradiation

It is well accepted that rapamycin inhibits mTORC1 signaling through binding with FKBP12. mTORC1 signaling plays crucial roles in regulating cell energy status and various cellular events, such as cell proliferation, apoptosis, differentiation, spermatogenesis, and tumorigenesis (Laplante and Sabatini 2012; Shimobayashi and Hall 2014; Li and Cheng 2016; Liu and Sabatini 2020). RpS6 is a protein translation mediator which can be activated by phosphorylation (Meyuhas 2015). From the results of the above part, it has been seen that radiation led to the decrease of spermatogonia and spermatozoa, which has an adverse effect on the normal and orderly spermatogenesis and the maintenance of testicular spermatogenic function. Several studies have shown that mTORC1 is involved in spermatogenesis and regulates the differentiation of spermatogonia (Busada et al., 2015; Suzuki et al., 2021; Wang et al., 2021). Therefore, we explore whether inhibition of mTORC1 can attenuate radiation-induced testicular damage.

**FIGURE 3:**
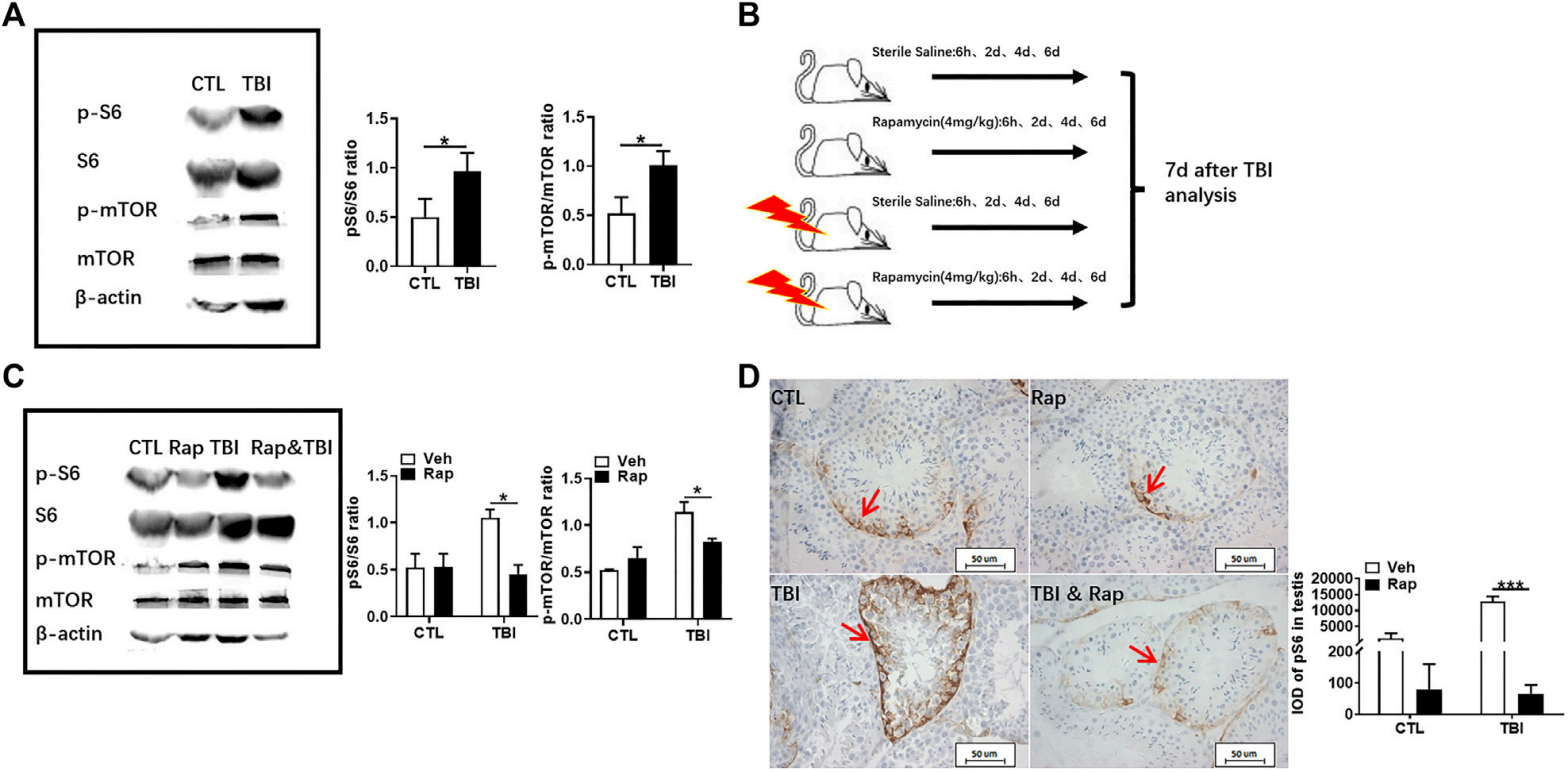
Rapamycin inhibits mTORC1 signal pathway under irradiation condition. **(A)** Expression of pS6, S6, p-mTOR, and mTOR in testis tissues was detected by western blotting at days 7 after irradiation. β-actin was used as a housekeeping control (left panel). The expression of pS6 and p-mTOR was quantitated using ImageJ software (right panel). **(B)** Experimental design of rapamycin. The mice were given rapamycin at the designed time after radiation, and the testis and epididymis of mice were taken 7 days after radiation. **(C)** Expression of pS6, S6, p-mTOR, and mTOR in testis tissues was detected by western blotting at days 7 after irradiation and rapamycin treatment. β-actin was used as a housekeeping control (left panel). The expression of expression pS6 and p-mTOR was quantitated using ImageJ software (right panel). **(D)** pS6 immunohistochemical staining. The expression of pS6 protein was analyzed 7 days after treatment to reflect the activation of mTORC1 pathway. Bar = 50 μm. The integrated OD (IOD) of pS6 was measured by image pro plus system and presented as mean ± SD (right panel). *p < 0.05, **p < 0.01, ***p < 0.001. Sidak’s multiple comparisons test was used after two-way ANOVA.

As shown in Figure 3A, Supplementary Figure S1, S2, they showed that the expression of phosphorylated S6 and mTOR was significantly increased in the testis of mice at 7 days after whole body X-ray exposure when compared to unirradiated mice. These data indicate that radiation can activate the mTORC1 signaling pathway in the testis. In order to further investigate the importance of mTORC1 activation in irradiation-induced testis damage, mice were irradiated and subsequently treated with rapamycin as shown in Figure 3B. Western blotting was used to assess whether rapamycin treatment inhibits mTORC1 signaling activation after irradiation. As shown in Figure 3C, Supplementary Figure S1, S2, the expression of phosphorylated S6 and mTOR proteins was significantly increased in the testes of mice exposed to radiation, which was significantly inhibited after rapamycin treatment. Meanwhile, pS6 was demonstrated through immunohistochemistry staining to reflect the status of mTORC1 signal pathway. Stained pS6 with brownish yellow localized at cytoplasm and mainly distributed in spermatogonia and primary spermatocytes, especially in spermatogonia. The results showed that the positive staining of pS6 in mouse testis significantly increased after radiation exposure (Figure 3D). The increased expression of pS6 in the mouse testis after irradiation can be inhibited by rapamycin treatment. These data indicate that radiation can damage mouse testicular tissue and activate mTORC1 signal pathway, which can be inhibited by rapamycin administration.

**FIGURE 4:**
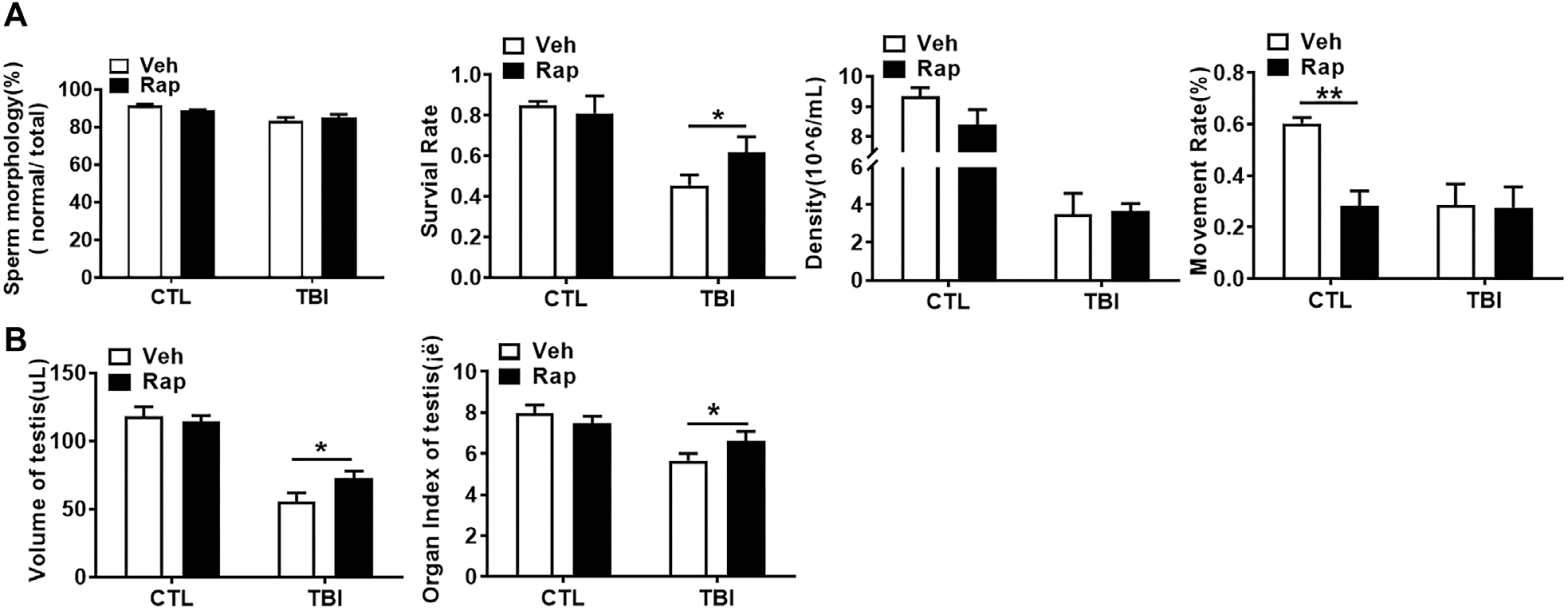
Rapamycin alleviates the decrease of sperm quality in Epididymis and testicular correlation coefficient caused by radiation. **(A)** Sperm quality parameters. From left to right, the sperm morphology, survival rate, concentration, and motility rate were shown. **(B)** Testicular volume and testicular organ coefficient in different groups. *p < 0.05, **p < 0.01, ***p < 0.001. Sidak’s multiple comparisons test was used after two-way ANOVA.

**FIGURE 5:**
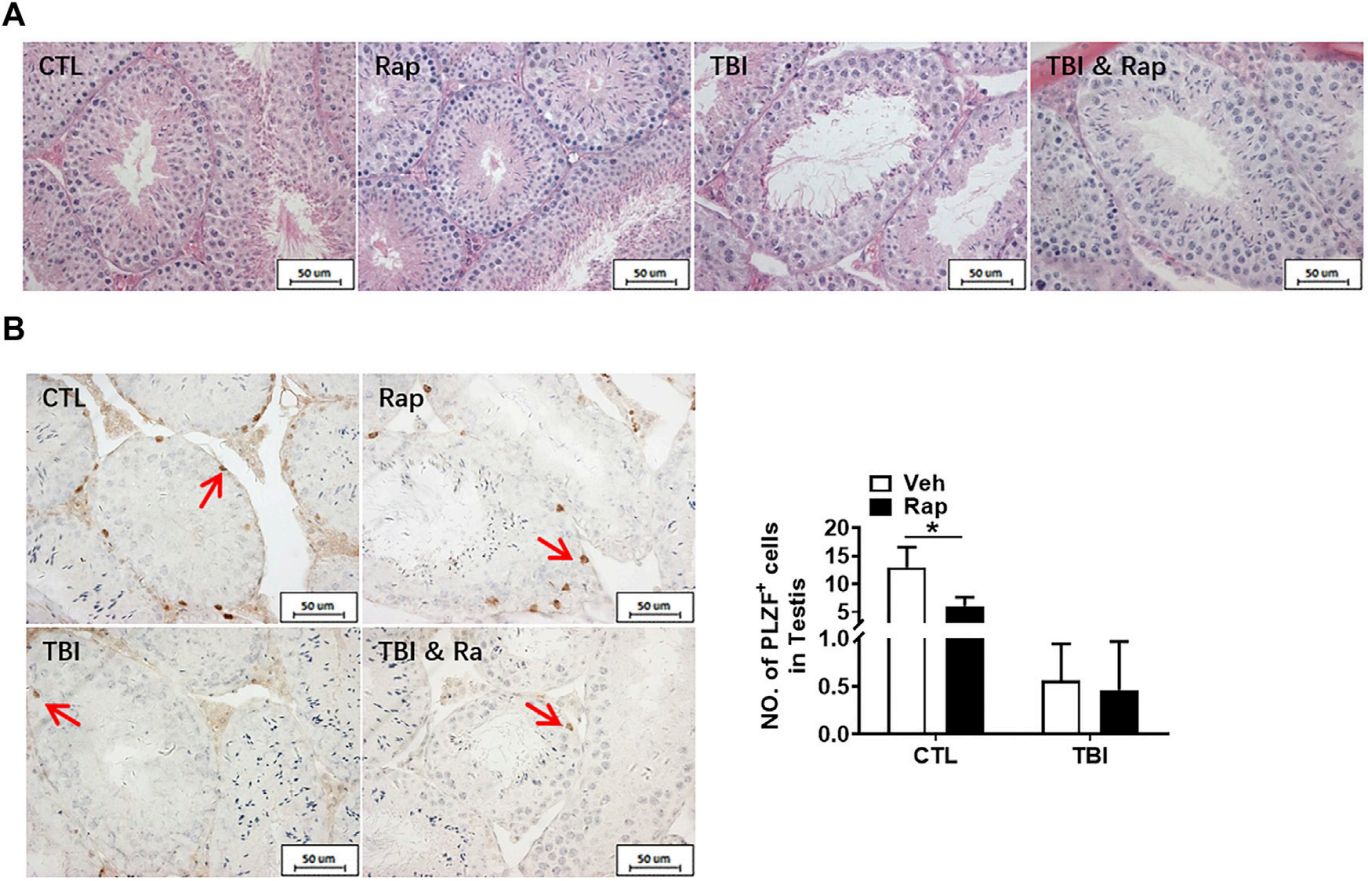
Rapamycin alleviates the testicular injury by radiation. **(A)** HE staining of testis. Bar = 50 μm. **(B)** PLZF immunohistochemical staining. PLZF immunohistochemical staining showed changes in the number of spermatogonia stem cells treated with different methods after radiation exposure (positive staining indicated with arrow). Numbers of PLZF + cells were counted under light microscope and expressed as mean ± SD (right panel). Bar = 50 μm *p < 0.05, **p < 0.01, ***p < 0.001. Sidak’s multiple comparisons test was used after two-way ANOVA.

To further examine whether inhibiting mTORC1 ameliorates radiation-induced testis injuries, the testicular coefficient, histopathological structure, spermatogonia number, and sperm quality were measured in different treatment groups. The indexes of sperm quality showed that the motility in rapamycin control group significantly decreased when compared to the non-irradiated group (Figure 4A). The sperm concentration and survival rate tended to decrease without statistically significant difference in the presence of rapamycin under non-radiation settings. Meanwhile, the sperm survival rate in the radiation + rapamycin group was significantly higher than that in the radiation exposure group while the other three indexes did not significantly change (Figure 4A). In addition, the results showed that the testicular volume and organ coefficient of mice significantly increased 7 days after whole body radiation exposure with rapamycin administration. These findings displayed that rapamycin could alleviate the adverse effects of radiation on the mouse testis (Figure 4B). As shown in Figure 5A, in comparison to irradiated testis, rapamycin treatment can significantly improve seminiferous tubule arrangement, intact basement membrane, regular lumen, and hierarchical spermatogenic cells. There was no significant difference in the histopathological structure of seminiferous tubules between the unexposed group and the exposed group in the presence of rapamycin (Figure 5A). By detecting the numbers of undifferentiated PLZF^+^ spermatogonia, we found that the numbers of undifferentiated PLZF^+^ spermatogonia in the rapamycin group were lower than that in the non-irradiated group (Figure 5B). However, rapamycin did not interfere with the numbers of spermatogonia after irradiation. Collectively, these results indicate that moderating mTORC1 signaling is beneficial to the maintenance of spermatogonia and spermatogenesis under radiation settings. It is suggested that rapamycin may ameliorate radiation-induced testicular injury by inhibiting radiation-activated mTORC1 pathway.

### Protective Role of Rapamycin on Testicular Tissue Injury Through Increasing Cell Proliferation Rather Than Inhibiting Apoptosis

**FIGURE 6:**
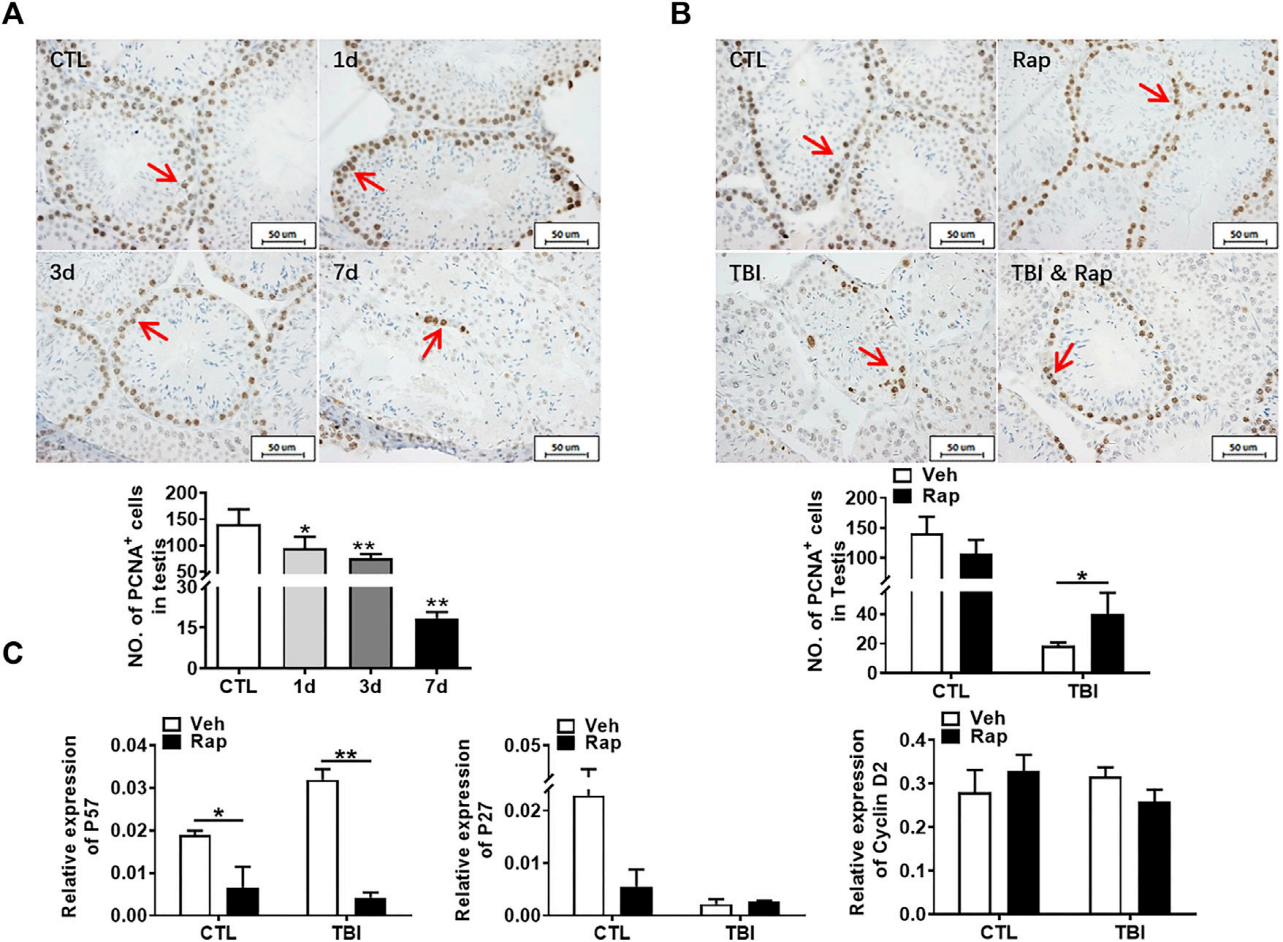
Rapamycin can alleviate the decrease of germ cell proliferation induced by radiation. **(A)** PCNA immunohistochemical staining. PCNA immunohistochemical staining reflects the changes of proliferating cells in mice at different time points after radiation exposure (positive staining indicated with arrow). Numbers of PCNA + cells were counted under light microscope and expressed as mean ± SD (middle panel). Bar = 50 μm *p < 0.05 vs. CTL. **p < 0.01 vs. CTL. ***p < 0.001 vs. CTL. Dunnett’s multiple comparisons test was used after one-way ANOVA. **(B)** PCNA immunohistochemical staining. PCNA immunohistochemical staining reflects the changes of proliferating cells in mice treated with different methods after radiation exposure (positive staining indicated with arrow). **(C)** Expression of P57, P27, and Cyclin D2. mRNA expression of P57, P27, and Cyclin D2 was conducted by QPCR. HPRT was used as a housekeeping gene. *p < 0.05, **p < 0.01, ***p < 0.001. Sidak’s multiple comparisons test was used after two-way ANOVA.

To explore the mechanism of radiation-induced testicular injury, proliferating cells were displayed with PCNA antibody through immunohistochemistry staining. The results showed that the numbers of proliferating cells in seminiferous tubules significantly decreased after radiation in a time-dependent manner (Figure 6A). There was no significant difference in cell proliferation between control group and rapamycin group under non-irradiation settings. Compared to the radiation group, rapamycin treatment increased the numbers of PCNA^+^ cells in irradiated mice (Figure 6B). Therefore, radiation-induced abnormal changes in the numbers of germ cells in the testis may be caused by the reduction of cell proliferation, which could be rescued by rapamycin treatment. In order to further verify the finding, RNA from mouse testis was extracted to examine the expression of P57, P27, and Cyclin D2 genes in testis by RT-PCR. The results showed that rapamycin administration decreased expression of P57 gene, which might promote cell proliferation in the presence of rapamycin (Figure 6C). However, there was no significant change in the expression of P27 and Cyclin D2 genes after irradiation in the absence and presence of rapamycin. These results imply that reduction of cell proliferation is involved in radiation-induced testis damage, which can be partially rescued by rapamycin treatment.

**FIGURE 7:**
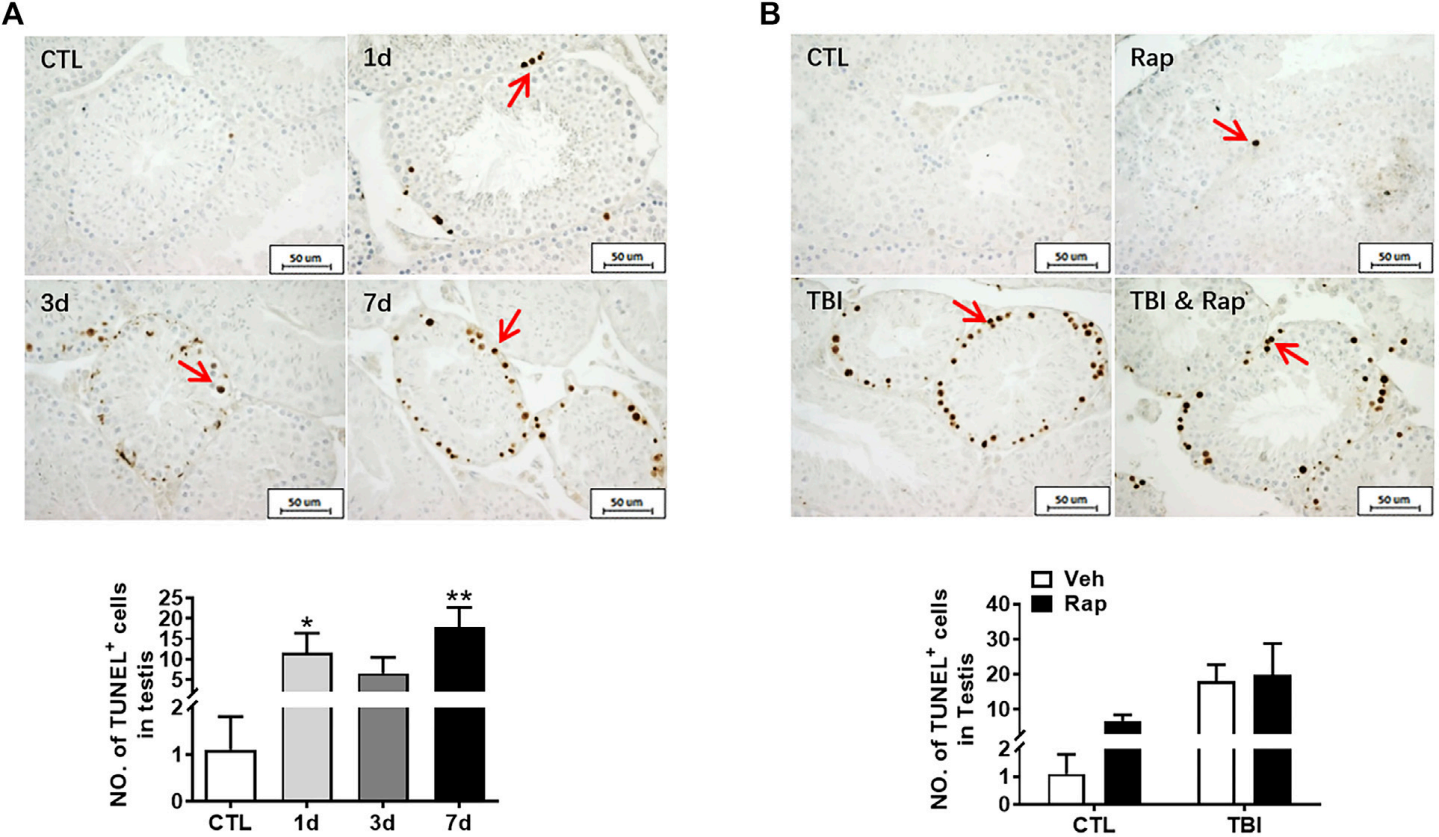
Rapamycin failed to alleviate the increase of apoptosis cells in testis induced by radiation. **(A)** TUNEL apoptotic staining. TUNEL apoptotic staining reflects the changes of apoptotic cells in mice at different time points after radiation exposure (positive staining indicated with arrow). Numbers of apoptotic cells were counted under light microscope and expressed as mean ± SD (middle panel). Bar = 50 μm *p < 0.05 vs. CTL. **p < 0.01 vs. CTL. ***p < 0.001 vs. CTL. Dunnett’s multiple comparisons test was used after one-way ANOVA. **(B)** TUNEL apoptotic staining. TUNEL apoptotic staining reflects the changes of apoptotic cells in mice treated with different treatments after radiation exposure (positive staining indicated with arrow). *p < 0.05, **p < 0.01, ***p < 0.001. Sidak’s multiple comparisons test was used after two-way ANOVA.

**FIGURE 8:**
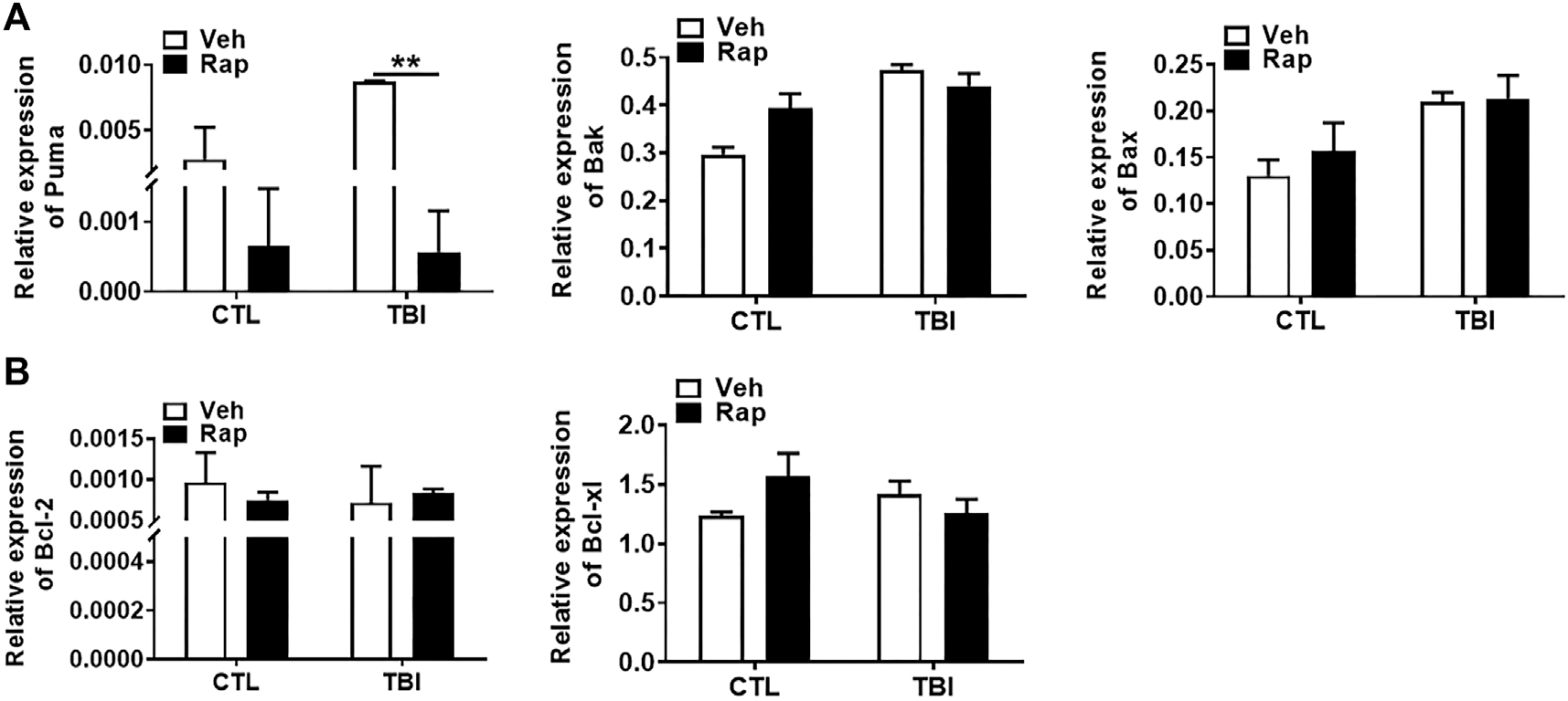
Rapamycin reduced the expression of pro-apoptotic gene Puma induced by radiation. **(A,B)** Expression of Puma, Bak, Bax, Bcl-2, and Bcl-xl mRNA was examined by QPCR. HPRT was used as a housekeeping gene. *p < 0.05, **p < 0.01, ***p < 0.001. Sidak’s multiple comparisons test was used after two-way ANOVA.

To further examine whether rapamycin could inhibit irradiation-induced cellular apoptosis, TUNEL apoptotic staining of germ cells was performed in the testis of mice at 1, 3, and 7 days after whole body radiation exposure. The results showed that the numbers of apoptotic cells in seminiferous tubules significantly increased after radiation exposure, especially on 7 days after radiation exposure (Figure 7A). To test whether rapamycin treatment alleviates radiation-induced cell apoptosis, the testicular tissues were examined at 7 days after radiation in the absence and presence of rapamycin. The results showed that rapamycin treatment failed to decrease the numbers of TUNEL^+^ cells in testis after radiation exposure (Figure 7B). We further examined the expression of pro-apoptotic genes (Puma, Bak, and Bax) and anti-apoptotic genes (Bcl-2 and Bcl-xL) in mouse testes by RT-PCR. The results showed that rapamycin could significantly reduce the expression of Puma in testis of mice after radiation (Figure 8A), but the expression of anti-apoptosis genes Bcl-2 and Bcl-xL had no significant change (Figure 8B). These results suggest that inhibition of Puma by rapamycin might be insufficient to inhibit the apoptosis of testicular cells after radiation. Taken together, rapamycin ameliorates radiation-induced testis damage mainly through regulating cell proliferation but not cell apoptosis.

### Effects of Rapamycin on Spermatozoa Production Under Irradiation

**FIGURE 9:**
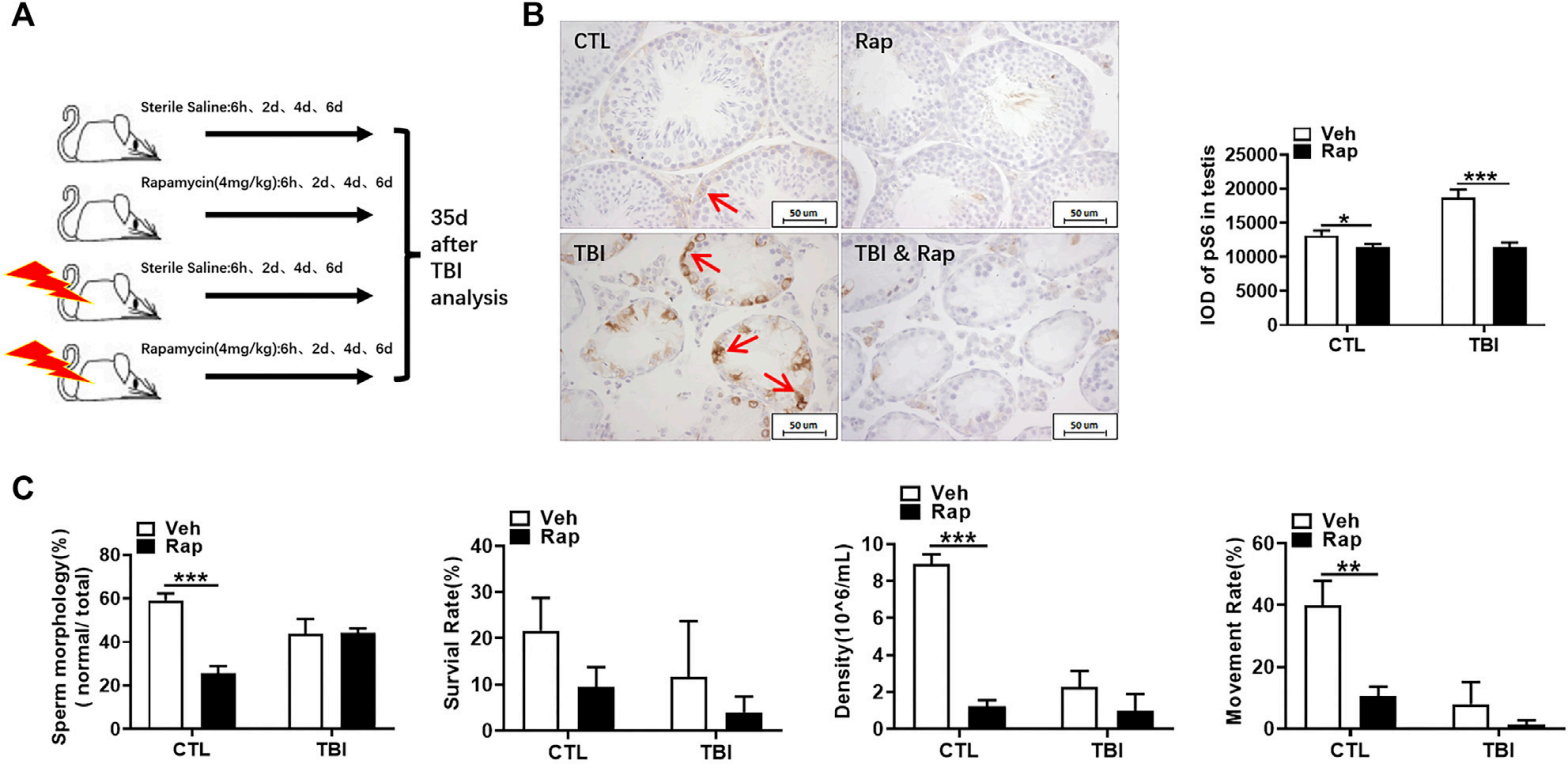
Rapamycin did not deteriorate the decrease of sperm quality 35 days after radiation. **(A)** Experimental design of rapamycin treatment 35 days after TBI. The mice were given rapamycin at the designed time after radiation, and the testis and epididymis were collected 35 days after radiation. **(B)** pS6 immunohistochemical staining. The expression of pS6 protein was analyzed 35 days after TBI to reflect the activation of mTORC1 pathway. Bar = 50 μm (positive staining indicated with arrow). The integrated OD (IOD) of pS6 was measured by image pro plus system and presented as mean ± SD (right panel). **(C)** Sperm quality parameters. From left to right, the sperm morphology, survival rate, density, and motility rate were shown. Bar = 50 μm *p < 0.05, **p < 0.01, ***p < 0.001. Sidak’s multiple comparisons test was used after two-way ANOVA.

It is known that it takes about 35 days from spermatogonia to spermatozoa through proliferation and differentiation. To investigate the effects of rapamycin on spermatogenesis cycle under physiological and irradiation conditions, mice were irradiated and subsequently treated with rapamycin at 6 h, day 2, day 4, and day 6 after the exposure. Testis and epididymis were analyzed at day 35 after irradiation as shown in Figure 9A. pS6 protein in testis was still highly expressed at 35 days after radiation, which was significantly decreased after rapamycin treatment (Figure 9B). To explore the effects of rapamycin on sperm in epididymis, rapamycin administration significantly increased sperm deformity rate and decrease sperm survival rate, density, and motility under physiological condition (Figure 9C). Thirty-five days after irradiation, sperm quality in epididymis was not remarkably improved after irradiation. Surprisingly, rapamycin did not deteriorate the decrease of sperm quality induced by radiation (Figure 9C). These data indicate that rapamycin alone has negative effects on spermatogenesis cycle under physiological condition. Rapamycin treatment did not further damage sperm quality during spermatogenesis cycle under irradiation condition while rapamycin administration accelerates the recovery of testis and epididymis in a short-term period after irradiation stress.

**FIGURE 10:**
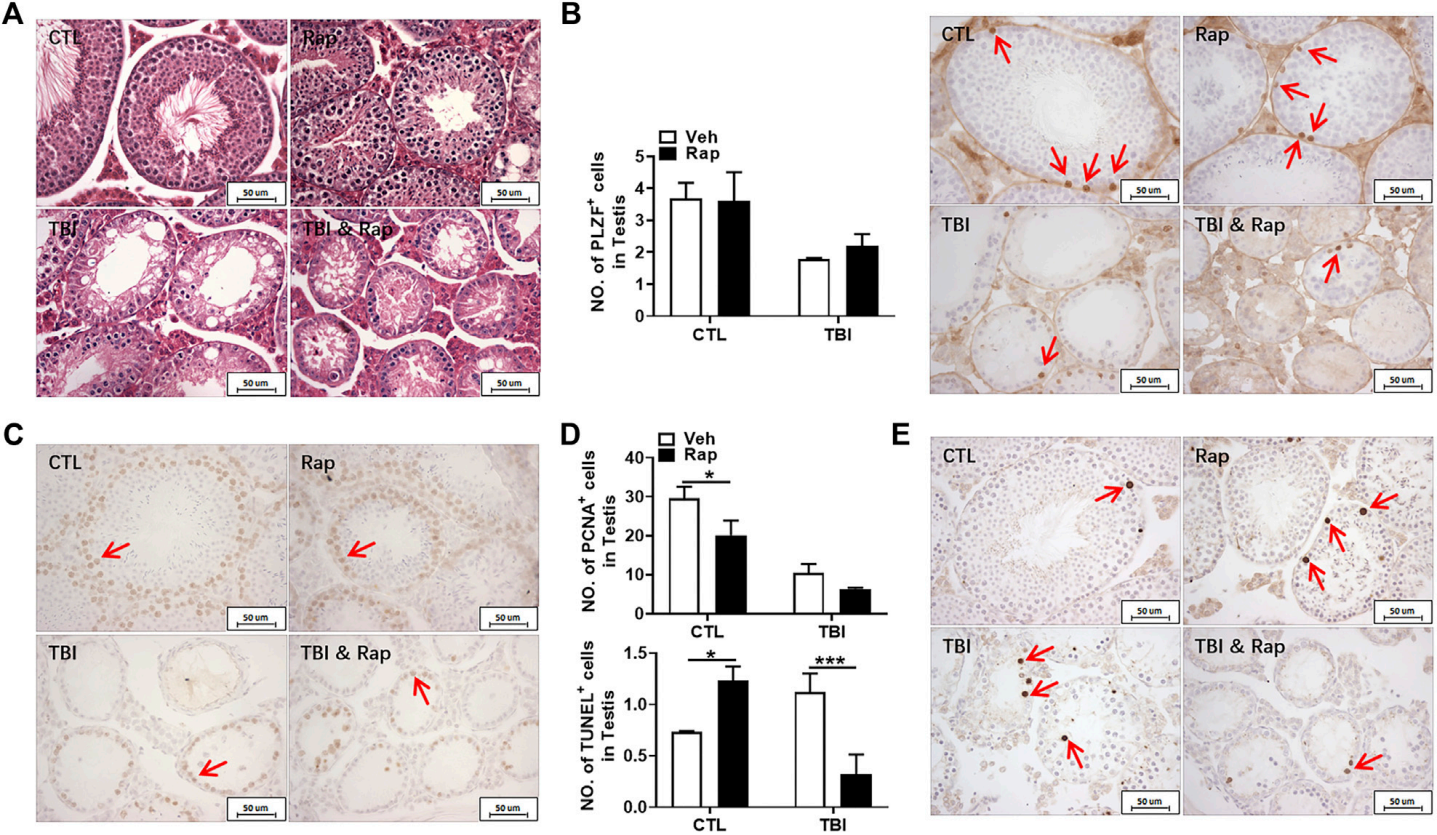
Rapamycin treatment benefited cell survival in testis after irradiation. **(A)** HE staining of testis. Bar = 50 μm. **(B)** PLZF immunohistochemical staining. PLZF immunohistochemical staining showed changes of numbers of spermatogonial stem cells in different groups 35 days after irradiation (positive staining indicated with arrows). Numbers of PLZF^+^ cells were counted under light microscope and expressed as mean ± SD (right panel). **(C)** PCNA immunohistochemical staining. PCNA immunohistochemical staining reflects the changes of proliferating cells in mice 35 days after radiation exposure (positive staining indicated with arrows). **(D)** Numbers of PCNA^+^ cells were counted under light microscope and expressed as mean ± SD (above panel). Numbers of TUNEL^+^ cells were counted under light microscope and expressed as mean ± SD (below panel). **(E)** TUNEL staining. TUNEL staining reflects the changes of apoptotic cells in mice after 35 days radiation exposure (positive staining indicated with arrows). Numbers of TUNEL^+^ cells were counted under light microscope and expressed as mean ± SD. Bar = 50 μm *p < 0.05, **p < 0.01, ***p < 0.001. Sidak’s multiple comparisons test was used after two-way ANOVA.

To further evaluate the effects of rapamycin on testis under physiological and irradiation conditions, HE staining was firstly used to assess testis morphological changes. As shown in Figure 10A, the structure of seminiferous tubules was integral in the unirradiated mice in the absence and presence of rapamycin. The testicular seminiferous tubules became loose in irradiated mice regardless of rapamycin administration. In the testis of irradiated mice, the diameter of seminiferous tubules became significantly smaller. The seminiferous epithelial cell layer became thinner, and the cells lost obviously. Moreover, there was no significant changes in the numbers of PLZF^+^ cells in non-irradiated testis 35 days after rapamycin treatment (Figure 10B). The numbers of PLZF^+^ cells in rapamycin-treated testis were mildly increased when compared to vehicle-treated testis at 35 days after irradiation (Figure 10B).

To assess the effects of rapamycin on cell growth and apoptosis in testis, PCNA and TUNEL staining were performed, respectively. Rapamycin treatment decreased numbers of PCNA^+^ cells in testis regardless of irradiation (Figure 10C). The numbers of TUNEL^+^ cells in rapamycin-treated testis were significantly increased when compared to vehicle-treated ones under non-irradiation condition (Figure 10D). However, the numbers of TUNEL^+^ cells in irradiated testis were significantly higher than those in non-irradiated testis, which was dramatically reduced after rapamycin treatment (Figure 10D). Collectively, although rapamycin treatment has negative effects on testis under non-irradiated condition, rapamycin can keep testis cell survival in a long-term period under irradiation stress condition, which might contribute to the recovery of reproductive ability and sperm quality.

## Discussion

Here, we have shown that X-ray whole-body irradiation can over-activate the mTORC1 signal pathway, which can be blocked by rapamycin. Rapamycin administration efficiently ameliorated radiation-induced testicular damage. In our previous studies, we have shown that radiation can over-activate the mTORC1 pathway in liver and kidney, leading to acute liver and kidney damage (Shao et al., 2018; Yang et al., 2019). When exploring the relationship between the level of oxidative stress and mTORC1 of adult stem cells including SSCs, researchers found that rapamycin can inhibit mTORC1 to alleviate the senescence of SSCs induced by oxidative stress and promote the self-renewal and proliferation of SSCs (Kofman et al., 2012), which is consistent with our finding that rapamycin can alleviate testicular damage caused by radiation.

Spermatogonia exhibits stage-dependent mTORC1 activity during postnatal development, which is highly active in differentiated spermatogonia and extremely low in undifferentiated spermatogonia. To test this difference, investigators conditionally deleted the mTORC1 inhibitor TSC1 and produced mutant mice that activated mTORC1 in a subset of undifferentiated spermatogonia. Depletion of TSC1 results in deficient testicular development, blocking spermatogenesis, decreasing numbers of germ cells, reducing sperm count, and dysfunctional fertility. Activation of mTORC1 promotes the differentiation of spermatogonia, damages the maintenance of germ lines, and leads to the early depletion of germ cells, thus harming spermatogenesis (Wang et al., 2016). Therefore, the balance between inhibition and activation of mTORC1 is critical for proper functional spermatogenesis.

In mammalian testis, the self-renewal, proliferation and differentiation of spermatogonia must be well coordinated. The imbalance of these processes will lead to infertility or tumor formation. mTORC1 acts as a central signal pathway in the self-renewal and balance of SSCs and is an intracellular homeostasis regulator. Various signaling pathways, including PI3K/AKT, ERK, MAPK and Wnt, are integrated in the regulation of spermatogenesis. At the same time, maintaining nutritional supply and cellular stress in the body can control cell growth, proliferation, differentiation, metabolism and autophagy (Laplante and Sabatini 2009). mTORC1 mediates the differentiation of spermatogonia induced by retinoic acid (RA). The effects of rapamycin lead to the accumulation of undifferentiated spermatogonia and hinders the orderly progress of spermatogenesis (Busada et al., 2015). Raptor, an associated protein of mTORC1, encodes an essential component of mTORC1. Raptor knockout mice do not have spermatogonia in adulthood along with loss of reproductive ability (Serra et al., 2019). It has been shown that the existence of moderate mTORC1 is necessary to maintain the normal function of testis (Suzuki et al., 2021). These data are consistent with the present results. Under unexposed condition, we found that the rate of sperm deformity in the rapamycin group was significantly higher than that in the non-irradiated control group. These data indicate that inhibition of mTORC1 has negative effects on sperm quality under non-irradiated conditions. These negative effects might be gradually recovered with the extension of time. It has been clinically reported that sirolimus, also known as rapamycin, was used as an immunosuppressant in a man receiving organ transplantation. Sperm quality of the man was significantly decreased, resulting in infertility. Surprisingly, male reproductive ability was recovered 12 months after sirolimus discontinuation (Deutsch et al., 2007). In animal experiments, it was also reported that rapamycin damaged male reproductive ability in a dose-dependent manner under normal physiological conditions (Kirsanov et al., 2020), but the testicular structure gradually returned to normal after 60 days of rapamycin withdrawal.

Spermatogenesis cycle from spermatogonia to spermatozoa is 35 days in mice (Griswold 2016). The numbers of spermatogonia stem cells in the rapamycin group have returned to the levels of normal mice in non-irradiated mice. The numbers of spermatogonia stem cells also tends to return to the normal levels at 35 days after irradiation and rapamycin treatment. The effects of rapamycin on irradiated testis may contribute to keep cell survival in that inhibition of mTORC1 pathway can enhance radiation resistance (Kahn et al., 2014). However, the long-term effects of rapamycin on non-irradiated and irradiated testis need to be further investigated in our future studies.

Germ cells are sensitive to radiation, which can induce apoptosis and negatively affect tissue structure and normal function (Zheng et al., 2018). Apoptosis was detected by TUNEL staining. We failed to detect that rapamycin decreased the apoptotic cell rate 7 days after radiation exposure even though Puma expression was significantly decreased in the presence of rapamycin. Elizabeth R had shown that inhibition of mTOR pathway can inhibit apoptosis (Sharlow et al., 2016), which is similar to our 35-days inhibition, indicating that rapamycin may have a protective effect by inhibiting apoptosis for a long time. PCNA is a helper protein of DNA polymerase necessary for DNA synthesis and a standard marker of proliferating cells (Aboul Fotouh et al., 2018). The damage of testis and the decrease of germ cells and spermatozoa caused by radiation are mostly related to the decrease of cell proliferation and the increase of apoptosis. Our current data showed that rapamycin treatment could increase cell proliferation in testis and significantly reduce the expression of P57 gene which inhibits cell proliferation. Therefore, radioprotection of rapamycin on testis might be mainly mediated by increasing cell proliferation.

The damage of X-ray whole-body irradiation to the body in mice may negatively affect multiple systems. Localized radiation may also inevitably affect the reproductive system. Testis is the direct organ to regulate reproductive function. Loss of reproductive function can be reflected by the testis injury. Currently, we are exploring comprehensive and detailed factors affecting reproductive function under irradiation setting, which benefits developing efficient countermeasures to protect reproductive system from radiation-induced injuries.

## Conclusion

In summary, X-ray whole body irradiation can activate mTORC1 signal pathway of mouse testis and cause testicular structural and functional damage. Rapamycin can inhibit the excessive activation of mTORC1 signal pathway and protect testis from testicular injury induced by X-ray whole body irradiation, which is achieved by promoting cell proliferation in testis.

## Data Availability

The original contributions presented in the study are included in the article/Supplementary Material, further inquiries can be directed to the corresponding author.

## Abbreviations

SSCs, spermatogenia stem cells; mTORC1, mammalian rapamycin target complex 1; pS6, phosphorylated S6; PLZF, promyelocytic leukemia zinc finger; GDNF, glial cell-derived neurotrophic factor; PCNA, proliferating cell nuclear antigen; Rap, rapamycin; BrdU, 5-bromo-2′-deoxyuridine; CTL, non-irradiated group; X, irradiated group; Rap, rapamycin group; X + Rap, irradiation + rapamycin treatment group; DAB, diaminobenzidine; mTOR, mammalian target of rapamycin; TUNEL, TdT-mediated dUTP nick-end labeling; IOD, integrated optical density.
