# Supplementary material for: Rapamycin Ameliorates Radiation-Induced Testis Damage in Mice
Source: Front Cell Dev Biol. 2022 Apr 25;10:783884. doi: 10.3389/fcell.2022.783884 (PMC9081527; doi:10.3389/fcell.2022.783884)
Supplement: Supplementary file 1 [file Image1.pdf]

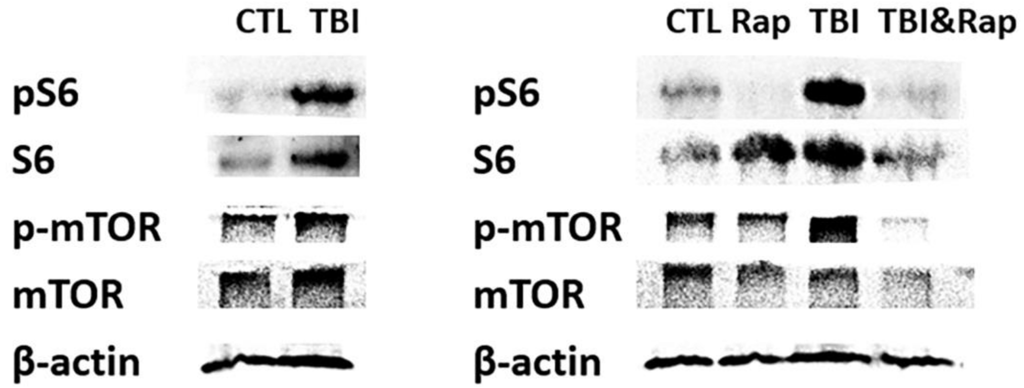

Supplementary Figure 1. Repeated experiment for main Figure 3A and C: expression of p-S6, S6, p-mTOR and mTOR after irradiation in the absence and presence of rapamycin.

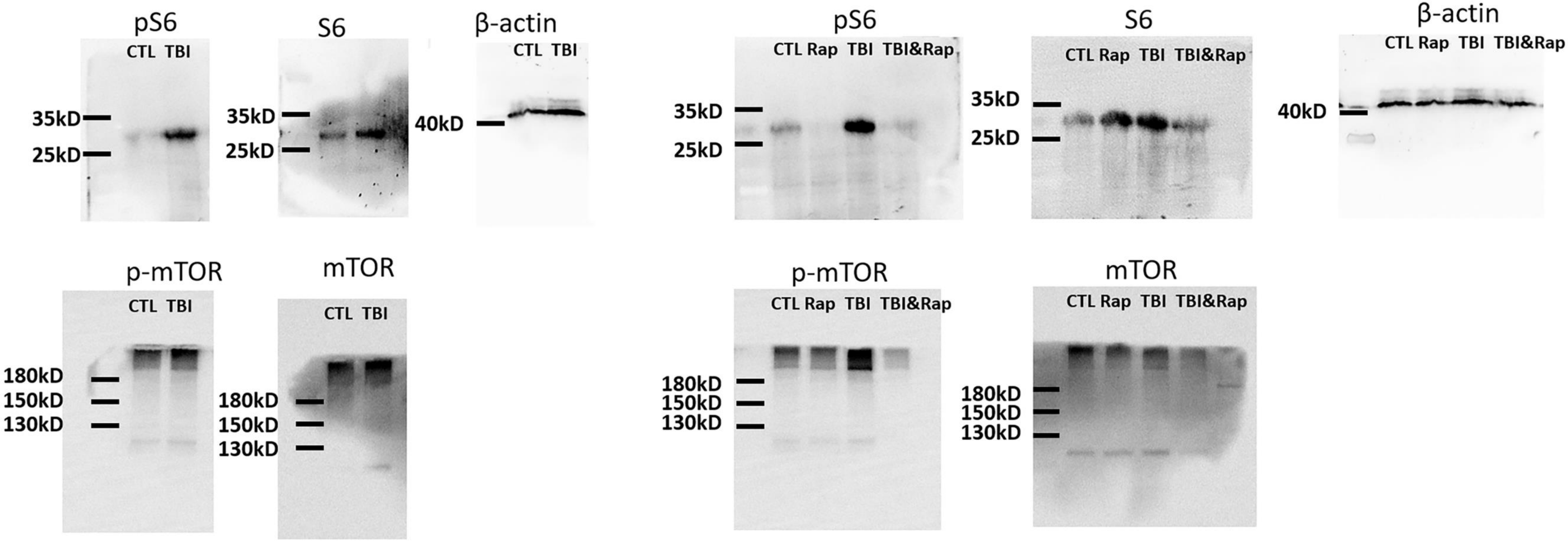

Supplementary Figure 2. Complete SDS-PAGE gels for panels shown in Supplementary Figure 1.
